# Supplementary material for: The influence of a quick educational video intervention on COVID-19-related knowledge in Ecuador
Source: PLoS One. 2023 Oct 5;18(10):e0291709. doi: 10.1371/journal.pone.0291709 (PMC10553301; doi:10.1371/journal.pone.0291709)
Supplement: Attachment — Submitted filename: Attachmnet.docx [file pone.0291709.s002.docx]

**TITLE: -** The Influence of a Quick Educational Video Intervention on COVID-19- related Knowledge in Ecuador

"This paper discusses the Influence of a Quick Educational Video Intervention on COVID-19- related Knowledge in Ecuador and the main contribution of the paper is comparing people ́s knowledge about COVID-19 before and after a brief video educational intervention.

**Recommendation**

"I recommend that this paper be accepted and I recommend that this paper be accepted after minor revision."

**Major comments:**

- The statistical analysis in this paper is suitable
- In terms of experimental technique, this paper is conventional/novel, and
- The Methods section does clearly explain
- The results obtained will be useful
- Some of the fundamental/recent papers in the field are not cited, among these…
- I would like to see some discussion of the findings of the papers in relation to recent findings and developments.

**Minor comments:**

It needs major grammatical revision
