# Supplementary material for: The influence of a quick educational video intervention on COVID-19-related knowledge in Ecuador
Source: PLoS One. 2023 Oct 5;18(10):e0291709. doi: 10.1371/journal.pone.0291709 (PMC10553301; doi:10.1371/journal.pone.0291709)
Supplement: Attachment — Submitted filename: Response to reviewers Impacto COVID Ed Paper.docx [file pone.0291709.s004.docx]

Rebuttal Letter

Dear Dr. Nour Amin Elsahoryi,

Thank you for considering our manuscript, "The Influence of a Quick Educational Video Intervention on COVID-19-related Knowledge in Ecuador," for publication in PLOS ONE. We are grateful for the feedback provided by the reviewers and have made several revisions in response to their comments. Below, we address each specific comment:

**Reviewer #1:**

**Conclusion based on statistical outputs:**

*Comment*: "Conclusion is not supported by the statistical outputs. For example, out of 28 chi-square tests, only 6 are significant."

*Response*:

*Thank you for your meticulous review and feedback on our research. We appreciate the time and effort you invested in understanding our work. We'd like to address the points you raised:*

1. *Levels of Significance: We understand that traditionally p<0.05 is considered the threshold for statistical significance. However, we believe that findings at p<0.1 may still offer valuable insights, especially in areas where the intervention might be showing a promising trend. While these may not be strictly significant, they could be indicative of a direction that warrants further investigation.*
2. *Real-world Implications: Regardless of the strict definitions of statistical significance, the areas that demonstrated change can have real-world implications. For instance, even a slight shift in understanding the incubation period or the contagiousness of COVID-19 can lead to better preventive behaviors in a community.*
3. *Interpreting Non-significant Results: We acknowledge that the majority of the areas did not show significant change. It's important to note that the absence of statistical significance doesn't necessarily imply the absence of change or the ineffectiveness of the intervention. Some areas might inherently remain stable post-intervention, or our sample size might not have been large enough to detect smaller yet meaningful changes.*
4. *Overall Impact: The intervention aimed to target specific misconceptions or gaps in knowledge. The areas where we observed significant changes indicate the effectiveness of our approach in certain domains. While we would have been elated to see statistically significant change across all areas, the observed changes in 6 (or 8, if considering p<0.1) areas still provide valuable insights into the potential impact of our intervention.*

We have revised the conclusion to ensure that it aligns with these comments.

**Chi-square test and conclusions:**

*Comment*: "However, the chi-square test does not support the statement in the conclusion."

*Response: With these revisions, our methodology is more explicit about the statistical approach and its rationale. The conclusion is also aligned with the results without overstating them.*

**Validation and reliability of the questionnaire:**

*Comment*: "How did you validate and assess the reliability of the questionnaire? It is very important."

*Response*: We have added a section in the Methods detailing the validation and reliability assessment of our questionnaire.

**Sample size and generalization:**

Comment: "Small sample size does not show any possibility of generalizing the tool practice on a mass scale."

*Response: We acknowledge this limitation and have included a statement in the Discussion section regarding it.*

**Alternative statistical tests:**

Comment: "Chi-square test is not the ideal test to show pre and post-intervention effect. If you can develop pre and post aggregated scores like mean values, then you can use a paired sample test for the purpose."

*Response: Thank you for your insightful comment regarding the choice of statistical tests. We appreciate your suggestion on utilizing a paired sample test based on pre and post aggregated scores like mean values.*

*Given the rapid and unprecedented nature of the COVID-19 pandemic, our team had to swiftly develop and deploy the questionnaire to capture the immediate perceptions and knowledge of the populace. As a result, the design of the questionnaire was oriented towards immediate understanding rather than creating a summative score. We acknowledge that, in an ideal scenario, having a summative score would have provided an additional layer of analysis and potentially allowed for the use of tests like the paired sample t-test.*

*However, we believe in the methodological integrity of our research processes. Making post hoc changes to generate aggregated scores could introduce biases or misinterpretations. Our choice to utilize the chi-square test, though not perfect, was driven by the nature of our data and the research context.*

*We hope our rationale clarifies the choices we made under the constraints and urgency of the situation. We truly value your feedback and will consider these insights in our future research endeavors.*

**Regression analysis:**

Comment: "Regression analysis will benefit to adjust the confounding effect."

*Response: Thank you for your recommendation regarding the implementation of regression analysis to account for potential confounding effects.*

*Upon your suggestion, we revisited our data and attempted regression analyses. However, we encountered several challenges that made this approach less ideal for our specific dataset:*

*Lack of Variation in Treatment: As you're aware, all participants received the same treatment, leading to no variation. This lack of variability is problematic for regression as it does not allow us to discern the treatment effect from other effects.*

*Sample Size Concerns: Our sample size, while sufficient for descriptive statistics and chi-square tests, is relatively small for regression analysis. Such a small sample could introduce biases, potentially leading to spurious or misleading results.*

*Nature of the Outcome Variable: Our outcome variables are categorical, with some having more than two categories. Regression models, especially with multi-category outcomes, require careful interpretation and larger sample sizes to ensure the stability and reliability of the estimates.*

*Given these challenges, we chose to remain cautious and decided against presenting potentially misleading results from the regression analysis. However, we have taken note of your valuable feedback and will be mindful of such considerations in future research, especially when designing studies with larger samples and varied treatments.*

*We hope our rationale provides clarity on our decision-making process. We truly value your insights and appreciate the thoroughness of your review.*

**Table one presentation:**

Comment: "Table one is not very user-friendly. Need to readjust."

*Response: We've modified the layout and presentation of Table 1 for better clarity.*

**Reference list:**

Comment: "I have not seen a reference list in the manuscript."

*Response: Our apologies for the oversight. We think there was an error when uploading the paper. We've added the reference list to the manuscript.*

**Abstract's statistical values:**

Comment: "The abstract needs more statistical values to show the results."

*Response: We have included more statistical values in the Abstract to provide a clearer picture of our results.*

**Reviewer #2:**

Comment: "Interesting, well-written, and well-designed review... Overall, a relook at the grammar and rephrasing of certain sentences is advised."

*Response: We appreciate your positive feedback. We have taken your advice and revised the manuscript for better clarity and grammar. We have rephrased most of the paragraphs to improve readability (considering we incorporated Reviewer #1´s corrections).*

*Journal Requirements:*

*We have also addressed the journal's specific requirements, including style requirements, grant information discrepancies, the role of the funders, data availability, the location of the ethics statement, and the completeness of our reference list.*

*We hope that our revisions address the reviewers' concerns. We kindly request you to reconsider our manuscript for publication in PLOS ONE. Enclosed are the revised manuscript with track changes, an and unmarked version of the paper.*

*Thank you for your time and consideration.*
